# The bZIP Transcription Factor Fgap1 Mediates Oxidative Stress Response and Trichothecene Biosynthesis But Not Virulence in *Fusarium graminearum*

**DOI:** 10.1371/journal.pone.0083377

**Published:** 2013-12-12

**Authors:** Mathilde Montibus, Christine Ducos, Marie-Noelle Bonnin-Verdal, Jorg Bormann, Nadia Ponts, Florence Richard-Forget, Christian Barreau

**Affiliations:** 1 Institut National de la Recherche Agronomique, Unité de Recherche 1264 MycSA, Villenave d’Ornon, France; 2 University of Hamburg, Biocenter Klein Flottbek, Department of Molecular Phytopathology and Genetics, Hamburg, Germany; 3 Centre National de la Recherche Scientifique, Unité de Recherche 1264 MycSA, Villenave d’Ornon, France; Seoul National University, Korea, Republic Of

## Abstract

Redox sensing is of primary importance for fungi to cope with oxidant compounds found in their environment. Plant pathogens are particularly subject to the oxidative burst during the primary steps of infection. In the budding yeast *Saccharomyces cerevisiae*, it is the transcription factor Yap1 that mediates the response to oxidative stress via activation of genes coding for detoxification enzymes. In the cereal pathogen *Fusarium graminearum*, Fgap1 a homologue of Yap1 was identified and its role was investigated. During infection, this pathogen produces mycotoxins belonging to the trichothecenes family that accumulate in the grains. The global regulation of toxin biosynthesis is not completely understood. However, it is now clearly established that an oxidative stress activates the production of toxins by *F. graminearum*. The involvement of *Fgap1* in this activation was investigated. A deleted mutant and a strain expressing a truncated constitutive form of *Fgap1* were constructed. None of the mutants was affected in pathogenicity. The deleted mutant showed higher level of trichothecenes production associated with overexpression of *Tri* genes. Moreover activation of toxin accumulation in response to oxidative stress was no longer observed. Regarding the mutant with the truncated constitutive form of *Fgap1*, toxin production was strongly reduced. Expression of oxidative stress response genes was not activated in the deleted mutant and expression of the gene encoding the mitochondrial superoxide dismutase MnSOD1 was up-regulated in the mutant with the truncated constitutive form of *Fgap1*. Our results demonstrate that *Fgap1* plays a key role in the link between oxidative stress response and *F. graminearum* secondary metabolism.

## Introduction

*Fusarium graminearum* (teleomorph *Gibberella zeae*) is the main causal agent of the Fusarium head blight (FHB), a disease that can affect wheat, barley, rice or maize, and potentially causes important economic losses. In addition, the occurrence of FHB is often associated with kernel contamination by mycotoxins that are produced in the field by *Fusarium*. Among the frequently encountered mycotoxins, type B trichothecenes (TCTB) are predominant [1]. Type B trichothecenes consist of deoxynivalenol (DON) and its acetylated C-3 and C-15 derivatives (3ADON and 15ADON, respectively), as well as nivalenol (NIV) and its C-4 acetylated derivative sometimes also called fusarenone X (4ANIV or FX) [2]. The presence of these mycotoxins in edible grains represents an important problem of food safety [3]. In Europe, maximum acceptable DON levels in food destined for human consumption were established (EC No1126/2007). Accordingly, grains exceeding the established limits are not permitted for commercialization for human consumption. Trichothecenes are heat stable molecules and are not degraded during current food processing [4]. As a result, the best way to restrict trichothecenes content in food is to limit their biosynthesis by the fungus before harvest. 

The genes involved in the type B trichothecenes biosynthesis pathway have been identified and named *Tri* genes [1,5,6]. Most *Tri* genes are clustered in the genome in a so-called *Tri5* cluster, named according to the gene coding for the first step of the biosynthetic pathway. It includes two transcription factors Tri6 and Tri10 [7]. These two proteins activate expression of other *Tri* genes [5,8]. It is however now clearly established that control of TCTB production is largely exerted by various transcriptional regulators known to regulate basal metabolic functions in fungi [9,10]. The full range of mechanisms controlling the accumulation of type B trichothecenes is, nonetheless, far from being completely understood. Indeed, during plant-pathogen interactions, pathogen recognition by the host leads to a plant-released oxidative burst, that consists of the production of reactive oxygen species (ROS) such as H_2_O_2,_ involved in processes related to plant defense mechanisms. In response to such an oxidative burst, pathogens induce oxidative stress response (OSR) mechanisms to scavenge the elevated ROS levels [11-13]. In addition, these ROS also play a role during fungal pathogenesis as demonstrated in the rice blast agent *Magnaporthe oryzae* [14]. The occurrence of an oxidative burst triggered after infection by *F. graminearum* has been demonstrated in barley or wheat [15,16]. Furthermore, previous works showed that treatment with H_2_O_2_ results in increased toxin accumulation and *Tri* genes expression by *F. graminearum in vitro* [17,18]. In that context, previous results suggested that the production of trichothecenes by *F. graminearum* could be a mechanism the fungus triggers to cope with ROS production by the plants [19]. 

The mechanisms underlying the regulation of TCTB production in the presence of H_2_O_2_ are largely unknown in *F. graminearum*. In yeasts, a large variety of transcription factors form a regulatory network that is activated by different oxidative stimuli such as peroxides and diamide [20,21]. Within this network, the key role of the well-known ap1 transcription factor Yap1, in response to oxidative stress has been largely documented in the yeast *Saccharomyces cerevisiae* [12,22]. Briefly, Yap1 localizes to the nucleus upon exposure to oxidative stress [23,24], where it activates the transcription of target detoxification genes, such as *cta1* and *ctt1*, coding respectively for peroxisomal and cytosolic catalase [25]. 

Yap1 homologues have been identified in several filamentous fungi. In the rice blast fungus *M. oryzae*, the homologue of Yap1, Moap1 mediates the response to oxidative stress and is essential for aerial growth, conidia formation and pathogenicity [14]. In *Aspergillus fumigatus*, a truncated version of the Yap1 homologue Afyap1 conferred resistance to oxidative stress [26]. In *Aspergillus parasiticus* and *Aspergillus ochraceus*, Apyap1 and Aoyap1 (the ap1 homologues in both species respectively) were not only involved in response to oxidative stress, but were also demonstrated to play a role in modulation of aflatoxin and ochratoxin A biosynthesis as well [27,28]. This last finding suggested a probable link between the response to oxidative stress and secondary metabolism. 

Here, we characterize for the first time *Fgap1*, a homologue of *Yap1*, in *F. graminearum*. 

We engineered genetically modified strains of *F. graminearum* lacking Fgap1 (*ΔFgap1* strain) or expressing a constitutively activated form of the protein (*Fgap1* ^*c*^ **_*trunc*_ strain) and showed the key role played by *Fgap1* in response to oxidative stress, in relation with the *in vitro* and *in planta* production of TCTB. 

## Materials and Methods

### Fungal strain and culture conditions

*Fusarium graminearum* INRA 605 (collection INRA from Bordeaux, France) of chemotype DON/15ADON was used throughout the study. In our culture conditions, 15ADON is predominantly detected. Wild-type strain and mutant strains were propagated on Potato Dextrose Agar (PDA, Difco-France) plates and maintained as frozen macroconidial suspensions at -80°C with 25% glycerol. 

Growth assays were performed on complete medium [29] supplemented prior solidification with H_2_O_2_, cadmium, sorbitol or sodium chloride (Sigma Aldrich®, Saint Louis, USA). 

Spore suspensions were generated by inoculating fifteen agar plugs in 150 mL of CMC medium (15 g/L carboxylmethyl cellulose, 1 g/L yeast extract, 0.5 g/L MgSO_4_.7H_2_O, 1 g/L NH_4_NO_3_, 1 g/L KH_2_PO_4_) [30] and incubating at 150 rpm and 25°C for three days. After filtration through Sefar Nitex 03-100 (100 µm, SEFAR AG - Switzerland), spores were counted on a Thoma cell and used as inoculum for GYEP liquid cultures (50 g/L glucose, 1 g/L yeast extract, 1 g/L peptone).

Ten mL of GYEP in 25 mL Erlenmeyer flasks were inoculated with 10^5^ spores and incubated in the dark, at 25°C and 125 rpm for up to 14 days in the presence or absence of 0.5 mM H_2_O_2_ (Sigma Aldrich®, Saint-Louis, USA). Cultures were done in triplicate. Cultures were harvested by centrifugation during 15 minutes at 4500 g and 4°C. Supernatants were stored at -20°C until trichothecene levels analysis. Mycelia were desiccated for 48h at 65°C to determine fungal dry biomass. When harvested for total RNA extraction, mycelia were quickly collected by vacuum filtration, rinsed with sterile water, and flash frozen in liquid nitrogen before short-term storage at -80°C until further analysis.

### Bacteria and yeast strains used for cloning

*Escherichia coli* XL1-blue strain (genotype *recA1 endA1 gyrA96 thi-1 hsdR17 supE44 relA1 lac* [F´ *proAB lacI*q*Z*Δ*M15* Tn*10* (Tetr)] and HST08 strain (genotype *F–, endA1, supE44, thi-1, recA1, relA1, gyrA96, phoA, Φ80d lacZΔ M15,* Δ (*lacZYA - argF*) *U169,* Δ (*mrr - hsdRMS - mcrBC*)*, ΔmcrA,* λ*–* ) (provided with the In-Fusion® HD Cloning Kit – Clontech) were used for plasmid propagation. *S. cerevisiae* FY1679 diploid strain (donated by François Doignon) of genotype MATa/MATα, ura3-52/ura3-52, trp1Δ63/TRP1, leu2Δ/LEU2, his3Δ200/HIS3, GAL2/GAL2 was used to perform the construction of the truncated gene.

### Type B trichothecenes analysis

Four ml of GYEP culture supernatant were extracted with 8 mL of ethyl acetate (Fisher Scientific, Waltham, USA). Five ml of the organic phase were then evaporated to dryness at 50°C under a nitrogen stream. For extraction from wheat samples, 5 volumes of acetonitrile/water (84%/16%) were added to finely grinded wheat kernels. After agitating for 1 hour, and centrifugation, 5 mL of the filtrate were purified using Trichothecene P columns (R-Biopharm, Darmstadt, Germany) before evaporation to dryness at 50°C under a nitrogen stream. 

Dried samples were resuspended in 500 µL of methanol/water (1:1, v/v) (Methanol Fisher Scientific, Waltham, USA) and cleaned up on 0.45µm filters (Phenomenex). Quantification analysis was performed on an Agilent Technologies 1100 series HPLC chain, equipped with an Agilent photodiode array detector (DAD) and the ChemStation chromatography manager software (Agilent, Waldbronn, Germany). Separation was achieved on a column kinetex 2.6U XB-C18 (4.6×150mm) maintained at 45°C. The mobile phase consisted of water acidified with orthophosphoric acid to reach pH 2.6 (solvent A) and acetonitrile (solvent B). The flow was kept at 1mL/min for a total run time of 27 min. The injection volume was set to 5 µL. TCTB were separated in gradient elution as follows: 93-7% B, 70-30% B in 10 min, 10-90% B in 25 min, 93-7% B in 12 min. UV-VIS spectra were measured from 220 to 550 nm and peak areas were measured at 230 nm. Quantification was performed using external calibration with standard solutions prepared from pure powders (Sigma Aldrich®, Saint-Louis, USA).

### *In silico* identification of Fgap1

The sequences of Yap1 from *S. cerevisiae* (NCBI accession number NP_013707.1) and its homologues in *Schizosaccharomyces pombe* (NCBI accession number NP_593662.1) and *A. fumigatus* (NCBI accession number XP_750882.1) were used as input for BLAST [31] against the *Fusarium* Comparative database from the Broad Institute (http://www.broadinstitute.org/annotation/genome/fusarium_group), to identify sequences with high similarities in the translated genome of *F. graminearum* (version 3). Multiple protein alignments were performed with ClustalW [32].

### Engineering of *ΔFgap1*, *ΔFgap1:ap1* and *Fgap1* ^*c*^_*trunc*_

*ΔFgap1* was obtained by replacing the genomic copy of the *Fgap1* coding sequence in the *F. graminearum* INRA 605 strain by a hygromycin resistance cassette. First, the flanking regions corresponding to a 988 bp fragment upstream the starting codon and a 800 bp fragment downstream the stop codon and the hygromycin resistance cassette were amplified using the Phusion High-Fidelity DNA polymerase (Finnzymes) as follows: the primers neoHY-1-fin F/neoHY-8-début R (see Table S1) were used to amplify a 2.1 kb fragment corresponding to the hygromycin resistance cassette, from the pBSCKneohygro plamid, containing *hph* gene under the control of the *Neurospora crassa CPC1* promotor and the *Aspergillus nidulans trpC* terminator. The flanking regions were amplified with two pairs of primers, YapAmontIFC F/ YAPAmont-HY R and YAPAval-HY F/ YapAvalIFC R respectively (see Table S1). PCR were performed using the following conditions: 98°C for 10 min, 35 × [98°C for 30 s, 64°C for 30 s, 72°C for 30 s/kb], 72°C for 5 min. The amplified fragments were purified from a 1% agarose gel using the QIAquick Gel Extraction Kit (Qiagen, Venlo, The Netherlands) according to the manufacturer’s instructions. The In-Fusion® HD Cloning Kit (Clontech, Mountain View, USA) was used for recombination of the three PCR products *via* overlapping hygromycin sequences and cloning into the pBCSK+ (Stratagene), according to the manufacturer’s instruction. The construction and sequence fidelity were confirmed by sequencing (Cogenics – Genome Express Grenoble – France). The construction was amplified using primers YapamF and YapavR and was used to transform protoplasts of the *F. graminearum* strain. The deleted strain selected for further studies was complemented by co-transformation with the wild-type sequence of *Fgap1*, including 1000 bp of the promotor region and 800 bp of the terminator region, and the pSM334 plasmid [33] carrying the geneticin resistance marker. 

*Fgap1* ^*c*^_trunc_ was generated in the yeast strain FY1679, maintained on solid YPD (yeast extract 10 g/L, peptone 20 g/L, dextrose 20 g/L). First, the *A. nidulans* promotor pGPD and the terminator TtrpC were amplified from the PAN8.1 plasmid [34] using the primer pairs 5fpGPDdeb F/pGPDyap R and TtrpC F/3rTtrpcfin R respectively (see Table S1). The *Fgap1* truncated version (protein truncated after the Q residue at position 517, see Figure S1) of the *Fgap1* coding sequence was amplified from the genomic DNA of the *F. graminearum* wild-type strain using the primer pair Yap-pGDP F/Yap-Ttrpc R (see Table S1). PCR was performed using the Phusion High-Fidelity DNA polymerase (Finnzymes) as follows: 98°C for 10 min, 35 × [98°C for 30 s, 62°C for 30 s, 72°C for 30 s/kb], 72°C for 5 min. The final construction was obtained by transforming the yeast strain FY1679 with the three PCR products and the pRS426 plasmid digested by *Bam*HI and *Hin*dIII, according to Collopy et al [35], allowing multiple recombinations *via* the overlapping sequences. The full-lengh construct was finally amplified by PCR using the primers GPDdeb F and Ttrpcfin R and cloned in the pCB1004, carrying the hygromycin resistance cassette [36] before transforming *F. graminearum* wild-type strain. The construction and sequence fidelity were confirmed by sequencing (Cogenics – Genome Express Grenoble – France).

### Fungal transformation

A total of 3×10^7^ spores were incubated in 100 mL of GYEP liquid medium for 12h at 25°C and 150 rpm in the dark. Cultures were then filtered on vacuum system, washed with 20 mL of NH_4_Cl 1M and treated with 16 mg of Driselase® (Sigma Aldrich®, Saint-Louis, USA) in a final volume of 20 mL of NH_4_Cl 1M. After 2 h of incubation at 26°C and 60 rpm, protoplasts were filtered through Sefar Nitex 03-100 (100 µm, SEFAR AG-Switzerland), collected by centrifugation at 950 g for 10 min and resuspended in 10 mL of 0.6 M Sorbitol in MOPS 10 mM, pH 6.3. The protoplast solution was then carefully layered onto 10 mL of a 1.2 M MgSO_4_ pH5.8. After centrifugation at 950 g for 10 min, purified protoplasts were collected at the phases interface and resuspended in 20 mL of sorbitol 1M in MOPS 10 mM pH 6.3. After centrifugation at 950 g for 10 min, the pellet was finally resuspended in 200 µL of sorbitol /CaCl_2_ buffer (Sorbitol 1 M, MOPS 10 mM, CaCl_2_ 40 mM, pH 6.3) and kept on ice until transformation. 

2×10^7^ protoplasts in 100 µL of sorbitol /CaCl_2_ buffer pH 6.3 were transformed with 5 µg of target DNA in 60 µL of Tris-EDTA-CaCl_2_ buffer (Tris 10 mM, EDTA 1 mM, CaCl_2_ 40 mM, pH7.5) and incubated for 20 min on ice. 160 µL of PEG 60% in Sorbitol 0.6 M was then added and incubation was carried on for 15 min at room temperature. Protoplasts were finally spread on PDAS agar plates (39 g/L Potato Dextrose Agar and sucrose 20%) containing hygromycin B (60 µg/mL) or geneticin (100 µg/mL). 

Transformants were purified by monoconidial isolation and screened for successful insertions of the desired constructs by PCR using the primers listed in Table S1. Genomic DNA was extracted from 50 mg of freeze dried mycelium as described previously [37]. Reactions were performed with the GoTaq DNA Polymerase (Promega, France) according to the manufacturer’s instructions. 

### Southern blot analysis

Transformants that passed the PCR pre-screening were further checked by Southern blot analysis, using the DIG system (Roche, Germany) according to a previously described protocol [38]. Five µg of genomic DNA from *ΔFgap1* or *Fgap1* ^*c*^_trunc_ was digested with SalI (Promega, France) or with PstI (Promega, France) respectively, and separated by 0.8% agarose gel electrophoresis. After transfer onto nylon membranes (Hybond N+, Amersham), DIG-labeled probes synthesized with a PCR DIG probe synthesis kit (Roche, Germany), were hybridized following manufacturer’s instructions. The probe 1 matches the core sequence of *Fgap1* and was generated by PCR amplification using the primers 5’-AATTCGGCAATGTCGATCTC-3’ and 5’-TGGGACAAGCTTGGAGTTTC-3’. The probe 2 corresponding to the 3’ end of *Fgap1*, was generated using the primers 5’-AACAGAACGGTGGACAATTC-3’ and 5’-TGTGCCCCACTTGATGACTA-3’. 

### RNA extraction and cDNA preparation

Fifty mg of freeze-dried mycelium was grinded with the TissueLyser® (Qiagen, Venlo, The Netherlands) for 2 min at 30 Hz, and total RNA was extracted using the RNeasy® Plant Mini Kit (Qiagen, Venlo, The Netherlands) following the manufacturer’s instructions. Eventual contaminations with gDNA were removed after treatment with the TURBO DNA-free™ Kit (Ambion) following the manufacturer’s instructions. The samples were quantified with the Nanodrop ND 1000 spectrophotometer (Labtech International, UK) and their quality was assessed by agarose gel electrophoresis. 1µg of total RNA was reverse transcribed using the SuperScript III First-Strand Synthesis System for RT-PCR (Life Technologies, Invitrogen) with oligo dT priming, according to the manufacturer’s instructions and kept at -20°C until further analysis. 

### Gene expression analysis

Analyses were performed using 1 µL of each cDNA preparation (corresponding to 10 ng of total RNA input in the reverse transcription) mixed in a 10 µL-reaction volume, using the QuantiFast™ SYBR® Green PCR kit (Qiagen, Venlo, The Netherlands). Reactions were carried out on a LightCycler® LC2.0 system equipped with the LightCycler software 3.5.3 (Roche, Germany). For each gene, efficiency was evaluated with serial dilutions of the pooled cDNA samples and data analyses were performed as previously described [17]. Expression levels of the studied genes (see Table 1), normalized to the expression of the housekeeping gene *GAPDH* were performed using REST® software (Relative Expression Software Tool) [39,40]. 

**Table 1:** Primers used to amplify cDNA by real-time PCR.

Gene (accession No)	Forward primer (5’ to 3’)	Reverse primer (5’ to 3’)	Tm(°C)
*GAPDH* (FGSG_06257)	TCAAGGGTGTTCTGGCCTAC	AGTAACCCCACTCGTTGTCG	60
*Fgap1* (FGSG_08800)	AACAGAACGGTGGACAATTC	TGATTGCAGTTCATGTTCTGT	65
*Tri4* (FGSG_03535)	TATTGTTGGCTACCCCAAGG	TGTCAGATGCGCCTTACAAA	60
*Tri5* (FGSG_03537)	GACCCTAAGCGACTACAG	GTGCTACGGATAAGGTTC	60
*Tri6* (FGSG_03536)	AGCGCCTTGCCCCTCTTTG	AGCCTTTGGTGCCGACTTCTTG	63
*Tri12* (FGSG_03541)	ATTCGGTACATGGGCT	GCAGTGCTGTTCGTCA	60
*Tri101* (FGSG_07896)	AGGGCATTAGCGAGGGAAACACA	ACGGCACGCCTTGGAGAGTAGAC	61
*Catalase 1* (FGSG_06554)	GGAAAGCCAGAACCAAACAA	CGTTGACGAGAGTGTAGGTGTTGA	58
*Catalase 2* (FGSG_02881)	TCTCTCCCTCCAACATGGTC	CAGGGTGCGTAGACTTGGTT	62
*Catalase 3* (FGSG_06733)	TCAACCGTCCTCTGTCTCCTATCC	AGCCTTTTCCTTGTGTCTGGTT	60
*MnSOD1* (FGSG_04454)	CTCTCCCCGATCTTCCCTAC	CGGGTAACGATGGACAGAGT	62
*MnSOD2* (FGSG_02051)	TTGCGTATCGTTACCACCAA	TCAACATAGGCAGCCTTTCC	60
*Cu/ZnSOD1* (FGSG_08721)	CACACCTTCGGTGACAACAC	GGTCTCGTCAGAAGGAGCAC	62
*Cu/ZnSOD2* (FGSG_00576)	AGCTCTCGCAGCAGTTCTTC	CAGGATCAACGTGAATGTGG	60

### Plant infection assays

The susceptible spring wheat cultivar Nandu (Lochow-Petkus, Bergen-Wohlde, Germany) was used for virulence assays. Plants were cultivated in a growth chamber at 20°C with a photoperiod of 16 h and 60% relative humidity, and then transferred to infection chambers with optimized conditions. For each strain tested, a suspension of 10^3^ conidia in 10 µl of the wild type and all mutants was inoculated into each of two central spikelets at the early stages of anthesis according to a previously described protocol [41]. The inoculated spikes were enclosed in small plastic bags misted with water for the first 3 days and then monitored for up to three weeks in the infection chambers. Wheat spikes inoculated with 10 µl of pure water were used as the negative control. After three weeks, the ears were separated from the whole plant; index values were determined by evaluating the proportion of infected spikelets for each spike and is given as the mean ± SD for all spikes. Spikes were then lyophilized, grinded and conserved at -20°C until toxin analysis. 

### Expression of results and statistical analysis

All results for trichothecene production were expressed as the arithmetic mean values ± SD of experiments performed in three independent biological repeats. Results for disease index were expressed as the arithmetic mean values ± SD of all spikes. Values were compared by performing the Student’s t-test. The value p = 0.05 was chosen as the point of statistical significance. Differential gene expression was analyzed with REST [39,40] using the gene encoding GAPDH as internal reference (5000 permutations, p ≤ 0.05). Results are reported in the log_2_ value of the ratio mutant *vs* wild-type strain or treated condition *vs* control. 

## Results

### Identification of Fgap1, a Yap1 homologue in *F. graminearum*

A genome-wide search of *F. graminearum* for the presence of a putative ap1 protein homologous to the S. *cerevisiae* Yap1, the *Schizosaccharomyces pombe* Pap1, and the *A. fumigatus* Afyap1 was performed using BLAST (see Materials and Methods). We identified the gene FGSG_08800 (Fusarium Comparative Database, accession number), coding for a 588 amino acids-long protein with significant homology with the above ap1 factors, referred to as *Fgap1* from now on. *Fgap1* is 2456 bp-long and contains two introns. Alignment of the Fgap1 protein sequence to those of Yap1, Pap1 and Afyap1 shows two conserved domains: an N-terminal b-zip domain (basic leucine zipper domain), and a C-terminal nuclear export signal (NES) embedded in a characteristic cysteine-rich domain, or c-CRD [42] (Figure S1). Yap1 and Pap1 classically contains another CRD, the n-CRD (Figure S1), that is absent from Fgap1. Previous data showed however that this n-CRD is optional for nuclear localization in *S. cerevisiae* [24] and the function of Fgap1 is likely unaffected by its absence. Fgap1 contains 3 others cysteines in the C-terminal part of the protein (Figure S1), whose function remains to be clarified. In conclusion, Fgap1 contains the bZip domain and the c-CRD domain containing the NES, essential for ap1-like full functions, and the corresponding nucleic sequence can be used for functional analysis of *Fgap1*. 

### Stress tolerance and *Fgap1*

**Figure 1:**
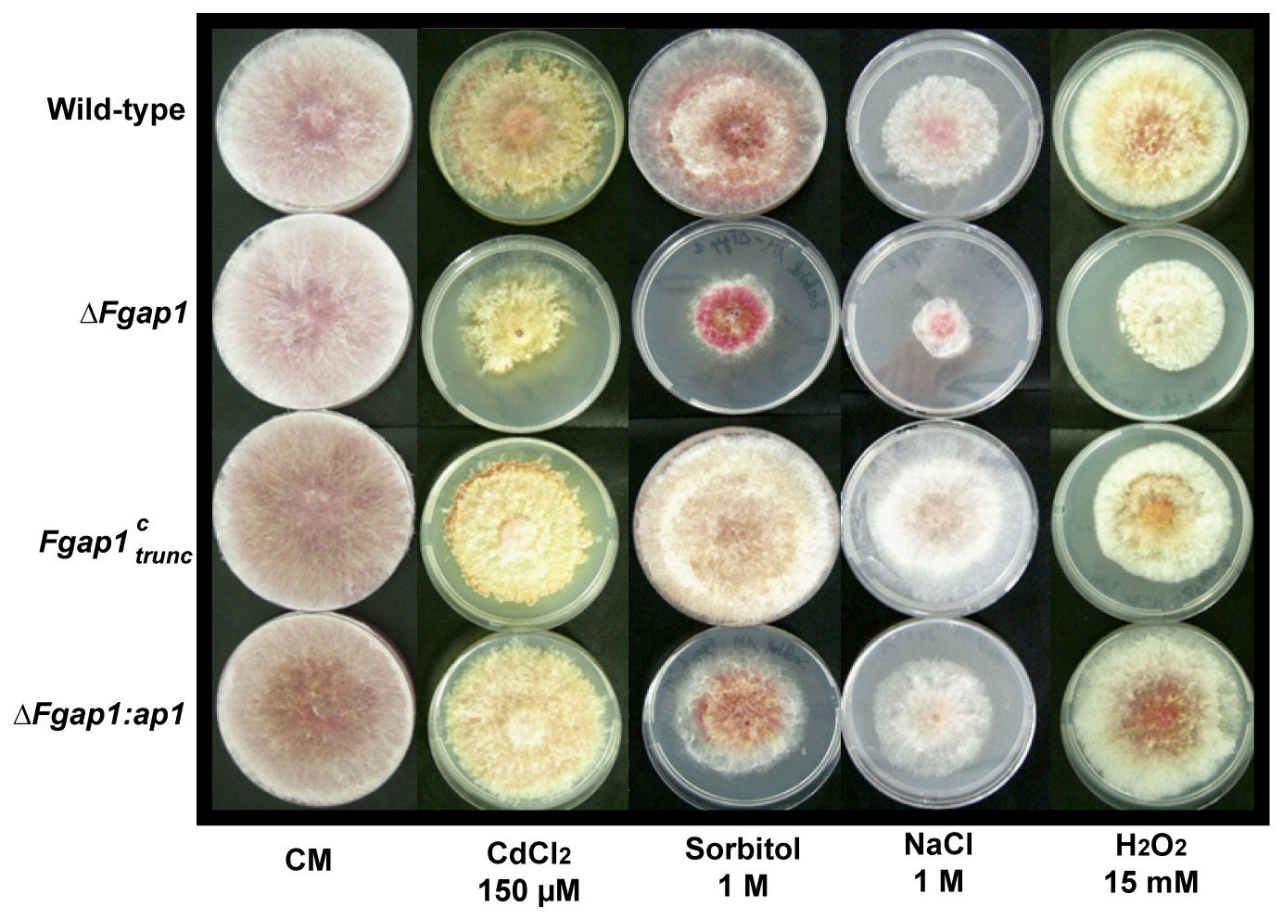
Radial growth assay to evaluate stress tolerance in *F. graminearum* wild-type strain and mutants. *F. graminearum* wild-type strain, *ΔFgap1*, *ΔFgap1:ap1*, and *Fgap1* ^*c*^_*trunc*_ were grown 7 days under oxidative stress with cadmium chloride 150 µM (C_d_Cl_2_) or H_2_O_2_ (15 mM), osmotic stress with sorbitol (1 M) or NaCl (1 M).

We analyzed the role of *Fgap1* in tolerance to stress, using reverse genetics approaches. Three different *F. graminearum* mutant strains were engineered. First, we deleted the genomic copy of *Fgap1* in *F. graminearum*, to create a loss-of-function mutant (*ΔFgap1*) (Figure S2). We verified using Southern blot that *Fgap1* was properly deleted and mono-copy (Figure S2). An intact copy of *Fgap1* including the promotor and the terminator regions, was subsequently re-introduced in the *ΔFgap1* strain (*ΔFgap1:ap1*) to restore its full functions. Finally, a truncated form of *Fgap1* lacking NES was constructed, therefore expected to be constitutively active (*Fgap1* ^*c*^_*trunc*_), and placed under the control of the constitutive *A. nidulans* promotor GPD and terminator TtrpC (see Figure S2) [36]. This form was introduced ectopically in *F. graminearum* wild-type strain. This construct was inspired by a previous study where a truncated version of *Yap1* was introduced in yeast [43] (Figure S2). We verified by Q-RT-PCR that *Fgap1* was not detected in the deleted mutant, overexpressed in the truncated mutant *Fgap1* ^*c*^_*trunc,*_ and expressed as wild-type levels in *ΔFgap1:ap1* mutant. In *M. oryzae*, such a deletion was shown to affect conidiation and radial growth [14]. Conidiation in liquid CMC medium of our mutants as well as growth of mycelia on CM plates after 4 days of culture were not affected (data not shown). Further, we tested *ΔFgap1*, *ΔFgap1:ap1*, and *Fgap1* ^*c*^_*trunc*_ mutants for tolerance to oxidative stress by H_2_O_2_ or cadmium, and NaCl or sorbitol-induced osmotic stresses (Figure 1). We compared the ability of the wild-type strain and the mutants to grow on agar complete medium supplemented with H_2_O_2_ 15 mM, NaCl 1 M, sorbitol 1 M, or cadmium 150 µM. Such concentrations did not affect substantially the growth of the wild-type strain after 7 days of growth. The growth of the deleted mutant *ΔFgap1* was however significantly affected. Even after seven days of growth the thallus was severely hampered and never reached the margin of the plate (Figure 1). Complementation restored the wild-type phenotype as shown with the *ΔFgap1:ap1* add-back strain. The growth of the *Fgap1* ^*c*^_*trunc*_ mutant was similar to that of the wild-type strain, except for sorbitol or NaCl-induced stresses where the *Fgap1* ^*c*^_*trunc*_ mutant exhibited a better adaptation. These results demonstrate that *Fgap1* is involved in the regulation of stress response in *F. graminearum*.

### Trichothecene production and *Fgap1*

**Figure 2:**
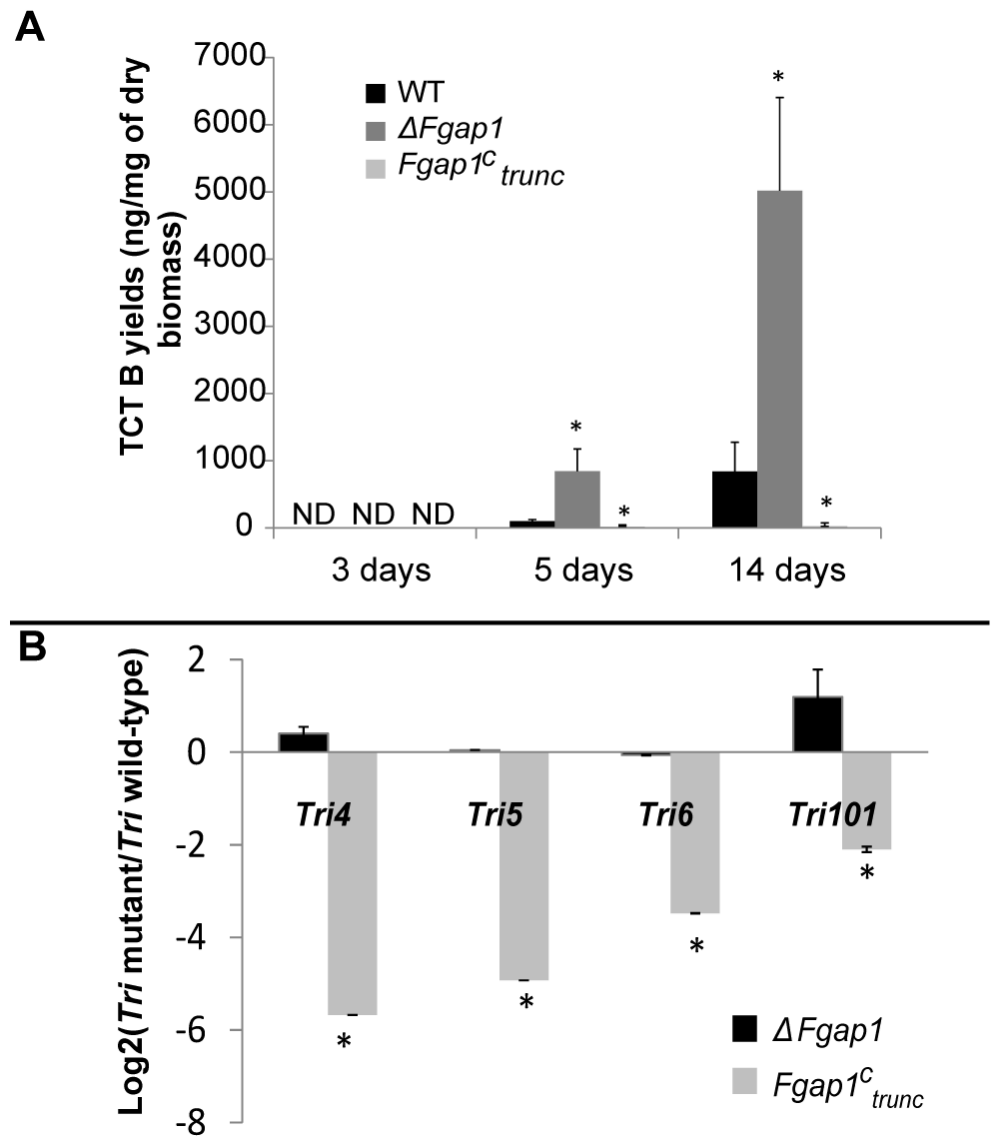
*Fgap1* plays a role in toxin accumulation and *Tri* genes expression. (A) DON + 15ADON yields (in ng of toxins produced per mg of dry fungal biomass) in the wild-type and the mutant strains after 3, 5 and 14 days of growth, ND stands for not detected. Error bars represent the standard deviation of three biological replicates. (B) Expression ratio mutant/wild-type (in log2 ratio scale) for *Tri* genes after 5 days of culture. The star indicates a significant difference compared to the wild-type (p<0.05).

In order to determine whether or not *Fgap1* is involved in DON and 15ADON production, the wild-type strain, the *ΔFgap1* and the *Fgap1* ^*c*^_*trunc*_ strains were cultured for up to 14 days and compared to control in terms of trichothecene production and *Tri* genes levels of expression (Figure 2). Toxins were detectable after 5 days of growth (Figure 2-A). The deleted mutant produced significantly more toxins than the wild-type strain at 5 days and at 14 days whereas the *Fgap1* ^*c*^_*trunc*_ mutant produced only traces amounts of trichothecenes, even after 14 days of growth. *Tri* genes expression was measured after 5 days of growth (Figure 2-B). Expression of *Tri 4* and *Tri 101* was slightly up-regulated in the *ΔFgap1* mutant compared to the wild-type strain and all the *Tri* genes were strongly down-regulated in the *Fgap1* ^*c*^_*trunc*_ mutant (Figure 2-B). These results strongly imply a role of Fgap1 in the regulation of trichothecene accumulation and *Tri* genes expression. 

In order to confirm the stimulating effect of the deletion of *Fgap1* on trichothecene accumulation, in another experiment, we compared the production of the *ΔFgap1:ap1* add-back strain to that of the *F. graminearum* wild-type strain and the *ΔFgap1* strain. The strains were then cultured for 4 days in GYEP liquid medium. Trichothecene (DON+15ADON) and *Tri5* gene expression levels were measured (Table 2). Again, toxin accumulation was strongly enhanced in the strain lacking *Fgap1*, with levels 20-fold higher than the wild-type strain. The complementation suppressed this strong increase, restoring a level of production in the same order of magnitude as the wild-type phenotype (Table 2). Concurrently, *Tri5* expression was up-regulated about 5-fold (log2 ratio of 2.3) in *F. graminearum ΔFgap1* strain compared to wild-type, consistent with toxin levels. A level of expression similar to wild-type was restored in the add-back strain *ΔFgap1:ap1* (Table 2). This result reinforces the role of *Fgap1* in the regulation of trichothecenes accumulation in *F. graminearum*.

**Table 2:** Toxin analysis and genes expression in the wild-type strain, the *ΔFgap1* and the *ΔFgap1:ap1* after 4 days of growth.

Analysis	Wild-type	Δ*Fgap1*	*ΔFgap1:ap1*
DON+15ADON yield (ng/mg of dry biomass)	10.4 ± 1.3	208.7 ± 90.5**^*a*^**	3.3 ± 2.1**^*a*^**
*Tri5* expression (log2(Mutant/Wild-type))**^*b*^**	_	2.3 ± 0.2**^*a*^**	0.9 ± 0.08

^a^ means significantly different from wild-type ; p≤0.05

^b^ Expression ratio mutant/wild-type in log2 scale

### Pathogenicity of *Fgap1* mutants

Both pathogenicity, i.e. the capacity to infect the inoculated spikelet, and virulence, i.e. the capacity to progress from one spikelet to the other ones below the inoculated point of the *Fgap1* mutant strains, were assessed using point inoculation on wheat. 

**Figure 3:**
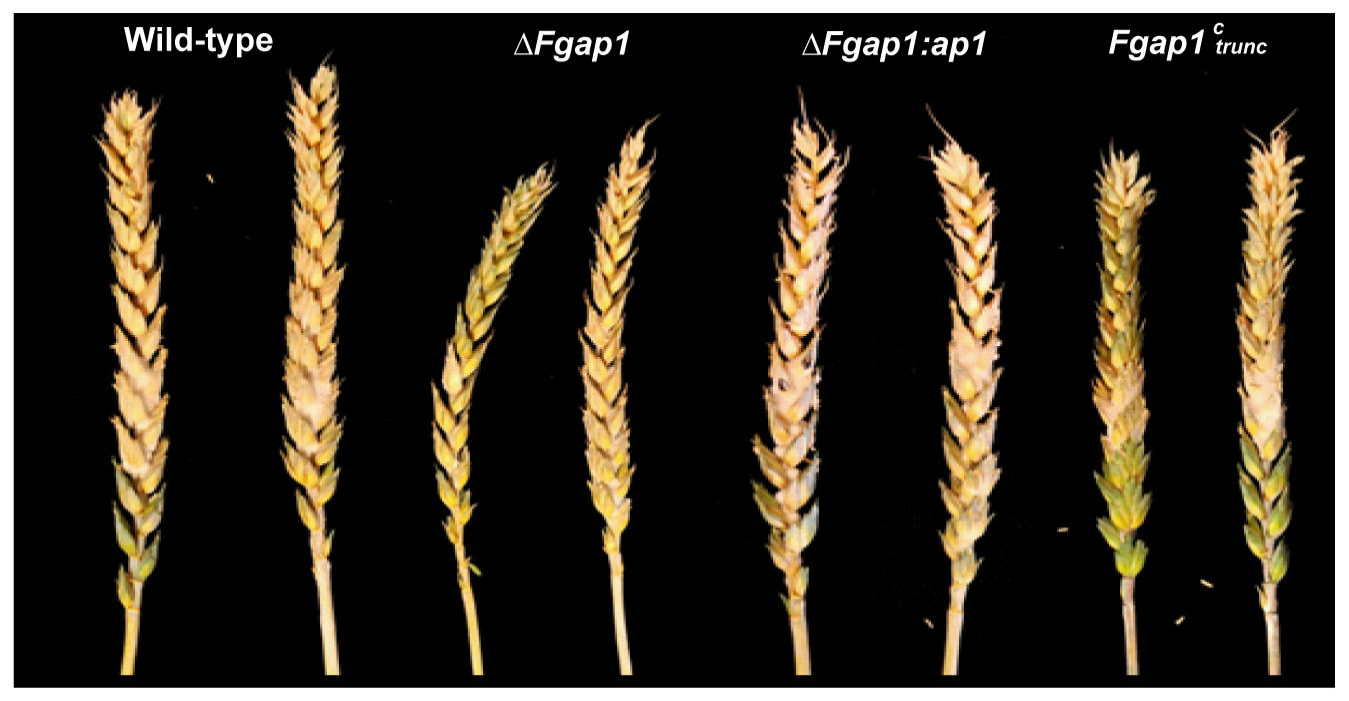
Wheat ears inoculated with *F. graminearum* wild-type strain, *ΔFgap1, ΔFgap1:ap1*, and *Fgap1* ^c^_trunc_. Wheat ears 21 days after point inoculation of two central spikelets. Bleaching indicates successful infection. Infection assays were performed with 10 replicates for each strain. For each strain, calculated disease index (% of infected spikelets) was: WT = 90.2 ± 25.6, *ΔFgap1* = 94.2 ± 11.1, *ΔFgap1:ap1* = 90.0 ± 22.4, *Fgap1* ^*c*^_trunc_ = 77.9 ± 25.2.

The wild-type strain, the *ΔFgap1* mutant and the add-back strain were pathogenic, caused serious bleaching, and presented similar disease index value (Figure 3). The *Fgap1* ^*c*^_*trunc*_ mutant showed a slightly reduced virulence, the spikelets at the bottom of the ears were not bleached and the disease index was slightly lower (Figure 3). However, due to high variability of the values measured for each spike, this difference was not significant. 

We further measured the levels of DON and 15ADON produced by each strain tested on the infected ears (Table 3). The wild-type strain and the *ΔFgap1:ap1* add-back strain produced similar levels of TCTB, meanwhile the deleted strain showed only a slight decrease in trichothecene accumulation. Moreover, the strain expressing the truncated *Fgap1* produced about three times less trichothecenes than the wild-type strain, consistent with the lesser virulence observed for this strain. Altogether, our results demonstrate that *Fgap1* is not essential for pathogenicity in *F. graminearum.*

**Table 3:** DON + 15ADON levels in wheat ears after 21 days after point inoculation of two central spikelets.

Strain	Toxin levels (µg/mg dry wheat)
Wild-type	153.4 ± 2.0
*ΔFgap1*	113.9 ± 8.1**^*a*^**
*ΔFgap1:ap1*	156.1 ± 1.3
*Fgap1* ^c^_trunc_	49.6 ± 10.7**^*a*^**

^a^ means significantly different from wild-type ; p≤0.05

### *Fgap1* and trichothecenes B production in response to H_2_O_2_ mediated oxidative stress

The wild-type strain, the deleted strain *ΔFgap1* and the strain expressing the truncated *Fgap1* ^*c*^_*trunc*_ were inoculated in liquid cultures supplemented or not with 0.5mM H_2_O_2_. Toxin accumulation was measured after 3, 5 or 14 days of growth and *Tri* genes expression were recorded after 5 days of culture. For each trial, treatment with H_2_O_2_ did not affect the accumulation of fungal biomass. In addition, supplementation with H_2_O_2_ did not affect pH compared to the not-supplemented control (data not shown). 

**Figure 4:**
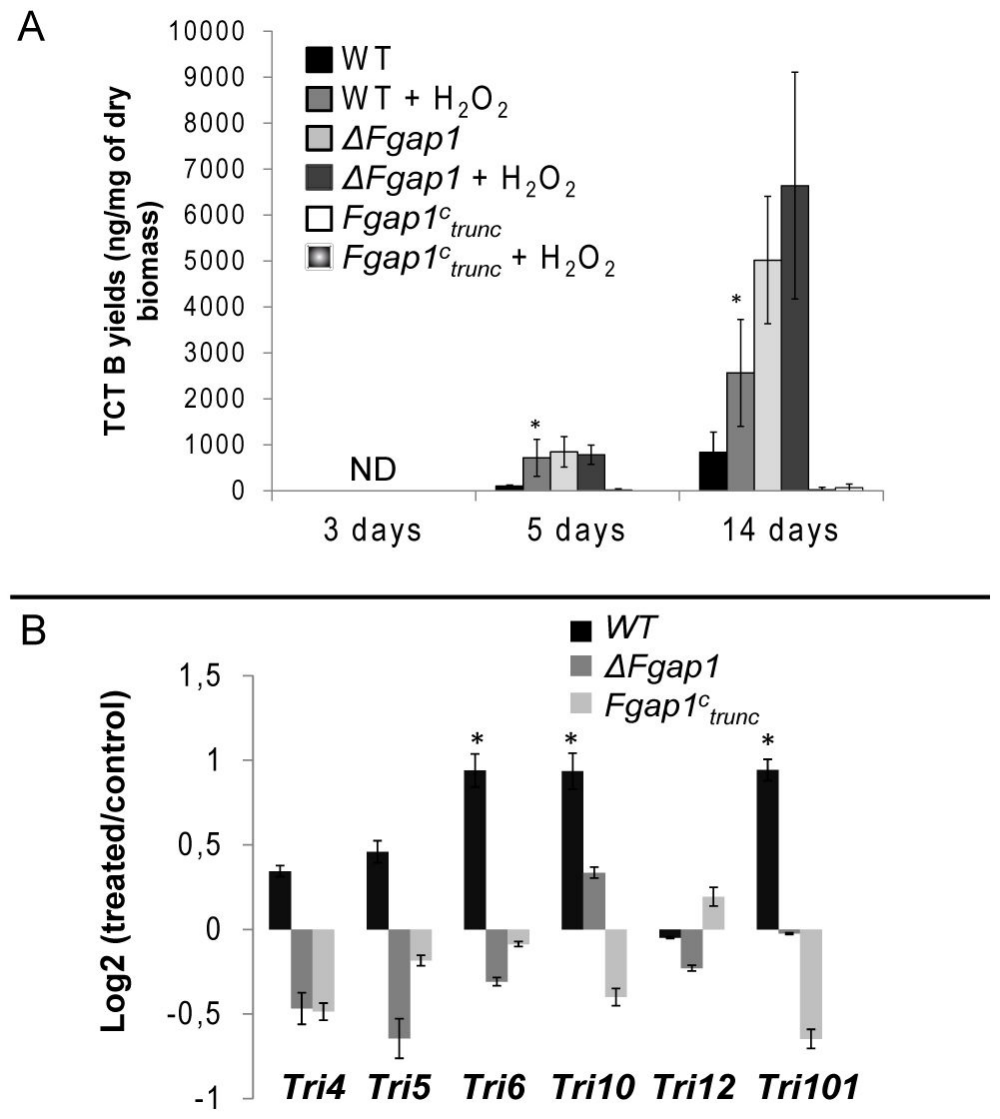
Toxin production and *Tri* genes expression in the wild-type strain and in the *ΔFgap1* and *Fgap1* ^c^_trunc_ mutants after exposure to oxidative stress by H_2_O_2_. *F. graminearum* wild-type or mutants were cultured up to 14 days in liquid medium supplemented or not supplemented with 0.5 mM H_2_O_2_. (A) DON + 15ADON yield (ng of toxins per mg of dry fungal biomass) after 3, 5 or 14 days of growth. Error bars represent the standard deviation of three biological replicates. The star indicates a significant difference for the treated condition compared to the control condition (p<0.05). (B) *Tri* genes expression (expression ratios treated/not treated in log2 scale) in 5 day-old cultures. *Tri4, Tri5, Tri6, Tri10, Tri12*, and *Tri101* were considered. The star indicates a significant difference of expression between treated *vs*. not treated (p<0.05).

DON and 15ADON were first detected after 5 days of culture in all cases (Figure 4-A). After 5 days of wild-type strain growth, TCTB accumulate seven times more in treated cultures than in control ones. After 14 days of growth, accumulation is still significantly enhanced in H_2_O_2_-treated cultures of *F. graminearum* wild-type (Figure 4-A). The tested *Tri* genes were broadly overexpressed in H_2_O_2_-treated wild-type cultures (Figure 4-B), including the gene *Tri101* localized outside the cluster *FgTri5*, consistent with higher toxin accumulation. The regulators *Tri6* and *Tri10* were significantly overexpressed in the presence of H_2_O_2_. These results are consistent with our previously published work [17]. In the absence of *Fgap1* (*ΔFgap1*), the toxin producing-activating effect of H_2_O_2_ previously observed for the wild-type strain was no longer visible (Figure 4-A), and *Tri* genes expression does not vary significantly upon treatment (Figure 4-B). On the contrary, the *Fgap1* ^*C*^_trunc_ strain, in which *Fgap1* is truncated and which produced only traces amounts of toxins in control condition, responded to H_2_O_2_ only with a slight increase in toxin accumulation (Figure 4-A) and no significant change in *Tri* genes expression was observed (Figure 4-B). All at once, these results demonstrate the strong role played by *Fgap1* in the regulation of toxin accumulation and *Tri* genes expression in response to oxidative stress, in *F. graminearum*. 

### *Fgap1* and genes encoding antioxidant activities

**Figure 5:**
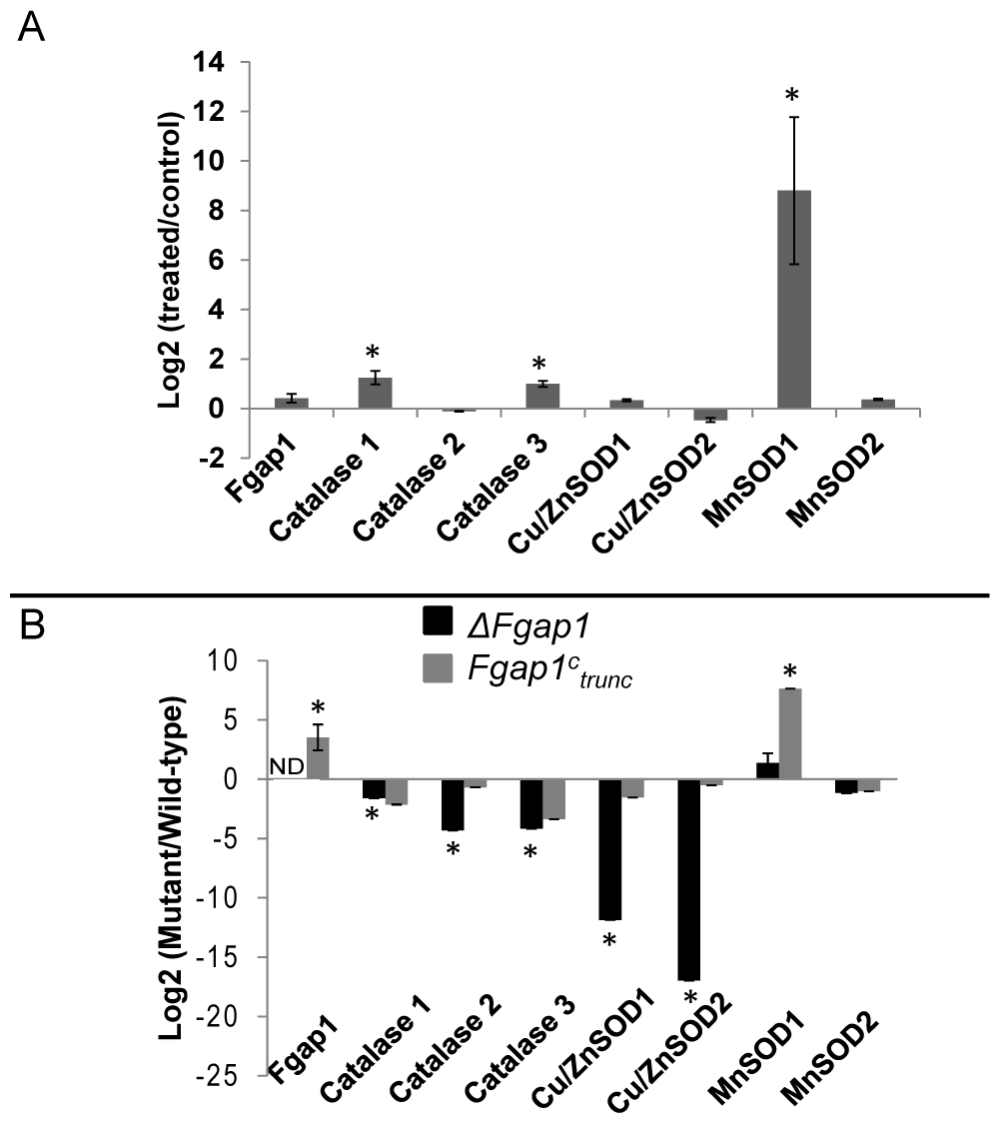
Expression of genes encoding antioxidant activities in the wild-type strain and in the *ΔFgap1* and *Fgap1* ^c^_trunc_ mutants. (A) Antioxidant genes expression (expression ratio treated/not treated in log2 scale) in the wild-type strain in 5-day old cultures. The star indicates a significant difference between treated *vs*. not treated (p<0.05). ND stands for not detected. (B) Antioxidant genes expression in the mutants strain compared to wild-type strain (expression ratios mutant/wild-type strain in log2 scale) in 5-day old cultures. The star indicates a significant difference for mutant compared to wild-type (p<0.05).

Our initial hypothesis postulates that *Fgap1* is a *Yap1* homologue, able to activate the expression of several genes encoding antioxidant activities required for detoxification upon oxidative stress. The level of expression of *Fgap1* in 5 days-old cultured mycelia was not affected by the treatment with 0.5 mM H_2_O_2_ (Figure 5-A). These observations suggest, that as in budding yeast, *Fgap1* may be regulated by stress-dependent nuclear localization rather than induced by oxidative stress [23]. We further considered the expression of seven genes (Table 1) encoding catalase or SOD activities (Figure 5), which are potentially under the control of ap1. These activities are involved in response to oxidative stress [12] and their expression was recently demonstrated to be Yap1-dependent in *A. ochraceus* [27]. The genes encoding the catalases considered in this study refer to the classification established in 2006 for fungal catalases. Catalase 1 corresponds to the spore-specific catalase, catalase 2 to a peroxisomal one, and catalase 3 to a secreted catalase [44]. Among SOD activities, two genes encoding putative MnSOD and two genes encoding putative Cu/ZnSOD were selected.

Three of the seven tested genes were significantly up-regulated under H_2_O_2_-induced oxidative stress in the wild-type strain (Figure 5-A): *Catalase1, Catalase3*, and *MnSOD1*, which showed the highest induction. These data indicate a direct involvement of these three genes in response to oxidative stress.

The expression of the same seven genes encoding antioxidant activities was then analyzed in the two *ΔFgap1* and *Fgap1* ^*c*^_*trunc*_ mutants and compared to the wild-type after 5 days of growth (Figure 5-B). In the deleted *ΔFgap1* mutant strain, the down-regulation of the three catalases and the two Cu/ZnSOD was consistent with an *Fgap1*-dependant expression (Figure 5-B, black bars). In the strain overexpressing the truncated *Fgap1*, a more than 10 time overexpression of *Fgap1* was observed compared to the wild-type as expected from an expression driven by the constitutive pGDP promotor (Figure 5-B, leftmost grey bar). Considering antioxidant genes*, MnSOD1* was the only one for which a clear overexpression was observed (Figure 5-B). These observations strongly support the role of *Fgap1* in the transcriptional regulation of genes encoding antioxidant activities. 

## Discussion

In this study, we provide for the first time the functional characterization of Fgap1 in *F. graminearum*. This protein is likely orthologuous to the previously characterized ap1 fungal proteins, such as the one identified in *S. cerevisiae* [22] or in *Aspergillus* species [27,28]. These ap1 fungal proteins were shown to play a key role in the plant oxidative stress response launched during a fungal pathogen infection process. Indeed, the generation of ROS, which is one of the earliest responses of the plant, is a defense mechanism in response of the recognition of the pathogen by the plant. Fungal pathogens have to induce an OSR mechanism to scavenge the elevated ROS levels and to be able to overcome the oxidative burst [11,13]. 

It is known in several fungi, that ap1 is one of the major regulators involved in activating OSR. *In S. pombe*, activation of genes encoding antioxidants activities, such as catalase, is mediated by ap1 [45]. In *Botrytis cinerea* and *A. ochraceus*, several antioxidant activities, such as catalase, superoxide dismutase or thioredoxin, are activated by ap1 [27,46]. Furthermore, the ap1 factor was demonstrated to be involved in the pathogenicity of *M. oryzae* or *Alternaria alternata* [14,47]. On the contrary, the factor has no impact on pathogenicity in *B. cinerea* [46] and in the foliar pathogen of maize *Cochliobolus heterostrophus* [48]. This demonstrates that ap1 proteins are required for full response to oxidative stress, but their role in virulence is divergent and suggests that other factors are redundant in this response to oxidative burst [49]. 

Here, we have, for the first time, identified *ap1* in *F. graminearum* by sequences homology with the ap1 proteins of *Aspergillus* spp. and yeasts. This gene, called *Fgap1*, was deleted and a truncated form was created to study the role of *Fgap1* in term of response to stress, trichothecene accumulation and pathogenicity in *F. graminearum*. 

We first compared the ability of the wild-type strain and the mutants to grow on complete mediums supplemented with H_2_O_2_, NaCl, sorbitol, or cadmium, to mimic several stresses. Our results suggested that in *F. graminearum, Fgap1* plays a role in response to these different stresses. Strikingly, the deleted mutant for *Fgap1* was only slightly sensitive to high levels of H_2_O_2_. This is different from the results observed for *Candida albicans* or *B. cinerea*, where the mutants were strongly affected in growth [43,46]. This suggests that in *F. graminearum*, other pathways of response to oxidative stress may compensate the lack of ap1 in response to H_2_O_2_. However, the deleted mutant for *Fgap1* was more sensitive to cadmium, NaCl and sorbitol. Concerning cadmium stress, our results are in full accordance with the observation that in *S. pombe* and *C. albicans*, deletion of *ap1* resulted in an increased sensitivity to cadmium [43,45]. Studies in *S. pombe* have demonstrated that overexpression of ap1 results in an increased expression of genes encoding ABC-type transporters. These proteins, when overexpressed, result in resistance to drugs [45,50]. These proteins could also be regulated by ap1 in *F. graminearum*. This could explain the phenotype of our deleted strain in response to cadmium. Furthermore, studies have demonstrated that in *S. pombe*, ap1 and Sty1 are part of the same signal transduction pathway. Sty1 is activated by MAPK pathways in response to numerous stresses [45]. This could explain the phenotype of the *F. graminearum* deleted *Fgap1* strain in response to osmotic stress. 

We previously showed that oxidative stress stimulated the accumulation of type B trichothecenes and *Tri* genes expression [17]. Here we clearly showed that *Fgap1* was involved in the regulation of toxin accumulation through modulation of *Tri* genes expression. The fact that the absence of ap1 resulted in an increase in toxin accumulation and *Tri* genes expression argue, however, in favor of an indirect effect. This is corroborated by the fact that the overexpression of the truncated form of *Fgap1* resulted in a strong down-regulation of *Tri* genes expression and toxin accumulation *in vitro*. These results correlate with those observed in *A. parasiticus* and in *A. ochraceus*, where a deleted strain for ap1 produced more toxins than the wild-type strain [27,28]. H_2_O_2_ was demonstrated to accumulate in the media during the culture of *F. graminearum* [51]. We can make the hypothesis that the *ap1* deleted mutant is unable to scavenge properly this excess of oxidant. Then, an increase of trichothecenes production, which consumes oxygen species, could represent an alternative way to reduce this excess [11]. 

In our results, the wild-type strain showed an overexpression of *Tri* genes and an increased level of trichothecene accumulation in response to oxidative stress. This response was lost in the deleted mutant. Expression of the truncated form of Fgap1 led to a strong down regulation of *Tri* genes and very few amounts of toxins even when an oxidative stress was applied. These results clearly demonstrated that *Fgap1* is involved in the regulation of trichothecene production in response to oxidative stress. The fact that overexpression of the truncated form strongly decreased the accumulation of trichothecenes suggests that the regulation by *Fgap1* is indirect. The truncation deletes the NES and is supposed to target *Fgap1* in the nucleus. If *Fgap1* was directly involved in *Tri* genes regulation, one could expect an increase of *Tri* genes expression. We can then raise the hypothesis that this *Tri* genes regulation operates via the control of the expression of one or various genes of which expression is controlled by ap1. 

It has already been demonstrated that ap1 was involved in the activation of OSR genes in several fungi [27,45]. *Catalase* expression and *sod* expression were especially reduced in an *A. ochraceus ΔAoyap1* mutant. As expected*, Fgap1* seems also strongly involved in the modulation of genes encoding antioxidant activities in *F. graminearum* as the absence of the gene resulted in a significant down regulation of the genes encoding catalase or sod activities. Surprisingly, for the truncated mutant, only one of these genes was strongly overexpressed. This gene encodes a putative MnSOD1. Others studies demonstrated that in *S. cerevisiae*, both ap1 and skn7 are necessary to activate genes involved in response to oxidative stress, via the NES of ap1. Here, the absence of the NES could impair the activation of OSR genes as the cooperation with skn7 become impossible [52]. This could explain why OSR genes are strongly down regulated when *Fgap1* is absent but not up-regulated when *Fgap1* is truncated. In order to explain the fact that the truncated version of *Fgap1* results in a strong overexpression of MnSOD1, we raise the hypothesis that this gene only requires ap1 for its activation and does not need the interaction with skn7. Blastp with this MnSOD1 showed that this sequence is homologous to the mitochondrial SOD of *S. cerevisiae*. Mitochondria are particularly sensitive to oxidative stress and play a central role in fungal cells [53]. This raises the hypothesis that regulation of the trichothecene pathway may be interconnected with the regulation of the mitochondrial metabolism of oxidative species. This observation is in agreement with the already described connection of secondary metabolism with mitochondrial metabolism [54,55]. 

Our results demonstrate that even if the *ap1* deleted strain produced more toxins than the wild-type strain *in vitro*, the deletion had no impact on pathogenicity and virulence *in planta*. This last result is consistent with results observed with *B. cinerea* or *C. heterostrophus* where the deletion of the ap1 factor showed no effect on virulence [46,48] but is opposite to the central role in virulence reported for *M. oryzae* [14]. This difference could come from the fact that the function of ap1 as pathogenicity factor is limited to biotroph pathogens [46].

To our knowledge, this is the first time that such a truncated form of ap1 was tested for pathogenicity. This mutant appears slightly less virulent and seems to produce lower levels of trichothecene *in planta* than the wild-type strain. It was demonstrated that trichothecene production in *F. graminearum* acts as an aggressiveness factor [56]. Therefore, the slight reduction in virulence we observed could result from the decrease in trichothecene production. Our result *in planta* differs slightly from the results obtained *in vitro* where the strain with the truncated ap1 consistently produced very low level of toxins. The difference observed between *in vitro* and *in planta* data could result from the occurrence of other compounds *in planta* such as agmatine, for example, that can stimulate toxin production and mask the effect of ap1 modification [57]. Trichothecene induction by plant compounds could then greatly hide the negative effect of the truncated ap1.

Our study demonstrates that the regulation of the trichothecene pathway is linked to the metabolism of oxidative species in *F. graminearum* and that *Fgap1* plays a central role in this regulation. According to our results, antioxidant activities controlled by Fgap1, such as mitochondrial MnSOD1, may be involved in OSR control, and could also play a key role in regulation of TCTB production. Further studies are needed to fully understand the role played by Fgap1 in the network involved in the regulation of response to stress and its link with secondary metabolism. 

## Supporting Information

Figure S1 **Multiple sequence alignment of Fgap1 with others ap1 homologous sequences.** Sequences from *F. graminearum* (FGSG_08800.3), *A. fumigatus* (XP_750882.1), *S. pombe* (NP_593662.1) and *S. cerevisiae* (NP_013707.1) were aligned with ClustalW online software. Conserved regions are shaded. Two domains are conserved: the b-zip domain, with the basic region and the leucine zipper and the nuclear export signal, embedded within the c-CRD. Blue arrows correspond to the cysteine residues of the c-CRD. Green arrows correspond to the n-CRD found in Yap1 and Pap1. Red stars correspond to the cysteine residues found only in the C-terminal part of Fgap1.
(DOCX)

Figure S2 **Construction of *ΔFgap1* and *Fgap1*^*c*^**_*trunc*_ mutants.** (A) Maps of *Fgap1wt* locus, the *ΔFgap1* construct containing the hygromycin resistance cassette and the *Fgap1* ^*c*^_*trunc*_ construction. * corresponds to the integrated codon stop. Bars represent labeled probes 1 and 2 used for the southern blot hybridization. For the *ΔFgap1* construct, among the 21 hygromycin resistant transformants, only 6 contained the replacement construct integrated at the *Fgap1* locus. The transformant number 15 was selected for complementation with the wild-type copy of *Fgap1*. Both transformant number 15 and one of the wild-type complemented transformants were submitted to a control by Southern blot analysis, using probe 1 or 2. Vertical black arrows stand for *Sal*I restriction sites. For the *Fgap1* ^*c*^_trunc_ construct, among 27 hygromycin resistant transformants, 5 contained the truncated construction. They were cultured in GYEP medium and after 11 days of growth, 4 produced few amounts of toxins. Overexpression of Fgap1 was analyzed by Q-RT-PCR. They were analyzed by southern blot using digestion with *Pst*I and the probe 2 to ensure that the construction was ectopic and to control the number of integrations. One transformant presented one integration and was selected for further experiments (data not shown). This transformant was analyzed by Q-RT-PCR analysis, confirming that *Fgap1* was overexpressed 10 times. This mutant was called *Fgap1* ^*C*^_*trunc*_ and retained for further experiments. (B) Southern blot hybridization. 1: pBCSK*ΔFgap1* (plasmid carrying the deletion construction); 2: wild-type strain; 3: Δ*Fgap1*; 4: Δ*Fgap1:ap1*. In the pBCSK*ΔFgap1*, probe 2 reveals a fragment of 3.9 kb when digested with *Bam*HI. In the wild type strain, probe 1 and 2 reveal a 3.3 kb fragment when digested with *Sal*I. In the *ΔFgap1* strain, probe 2 reveals a 4.8 kb fragment when digested with *Sal*I. In the complemented strain *ΔFgap1:ap1*, probe 1 and probe 2 reveal a fragment of unknown size due to ectopic integration of the wild-type copy of *Fgap1* when digested with *Sal*I.
(DOCX)

Table S1 **List of primers used for vector constructions and transformants screening.** ^a^Capitalized sequences in the YAPAmont-HY R and YAPAval-HY F primers correspond to the hygromycin resistance cassette to allow overlapping between the 5’UTR, the hygromycin resistance cassette and the 3’UTR. ^b^Sequences underlined in the YapAmontIFC F and YapAvalIFC R primers correspond to the multiple cloning site of the pBCSK plasmid digested with *BamH*I. ^c^Sequences underlined in the 5fpGPDdeb F and 3rTtrpcfin R primers correspond to the multiple cloning site of the pRS426 plasmid digested with BamHI and HindIII. ^d^Capitalized sequences in the Yap-pGPD F and Yap-TtrpC R primers correspond to pGPD and TtrpC sequences respectively to allow overlapping between pGPD, *Fgap1* and TtrpC.
(DOCX)
