# Supplementary figures and images for: The bZIP Transcription Factor Fgap1 Mediates Oxidative Stress Response and Trichothecene Biosynthesis But Not Virulence in Fusarium graminearum
Source: PLoS One. 2013 Dec 12;8(12):e83377. doi: 10.1371/journal.pone.0083377 (PMC3861502; doi:10.1371/journal.pone.0083377)

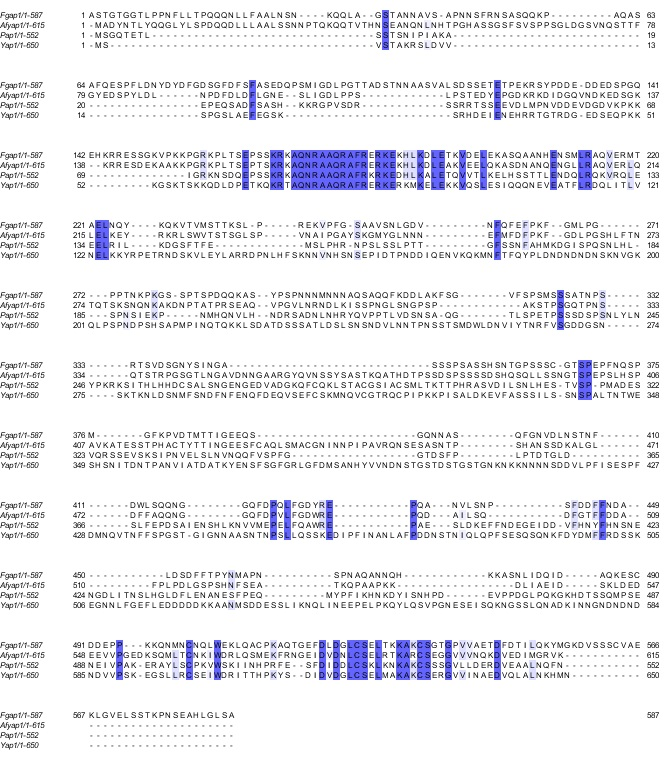


Nuclear export signal

b-zip domain

Figure S1

Supplement: Figure S1 — Multiple sequence alignment of Fgap1 with others ap1 homologous sequences. Sequences from F. graminearum (FGSG_08800.3), A. fumigatus (XP_750882.1), S. pombe (NP_593662.1) and S. cerevisiae (NP_013707.1) were aligned with ClustalW online software. Conserved regions are shaded. Two domains are conserved: the b-zip domain, with the basic region and the leucine zipper and the nuclear export signal, embedded within the c-CRD. Blue arrows correspond to the cysteine residues of the c-CRD. Green arrows correspond to the n-CRD found in Yap1 and Pap1. Red stars correspond to the cysteine residues found only in the C-terminal part of Fgap1. (DOCX) [file pone.0083377.s001.docx]

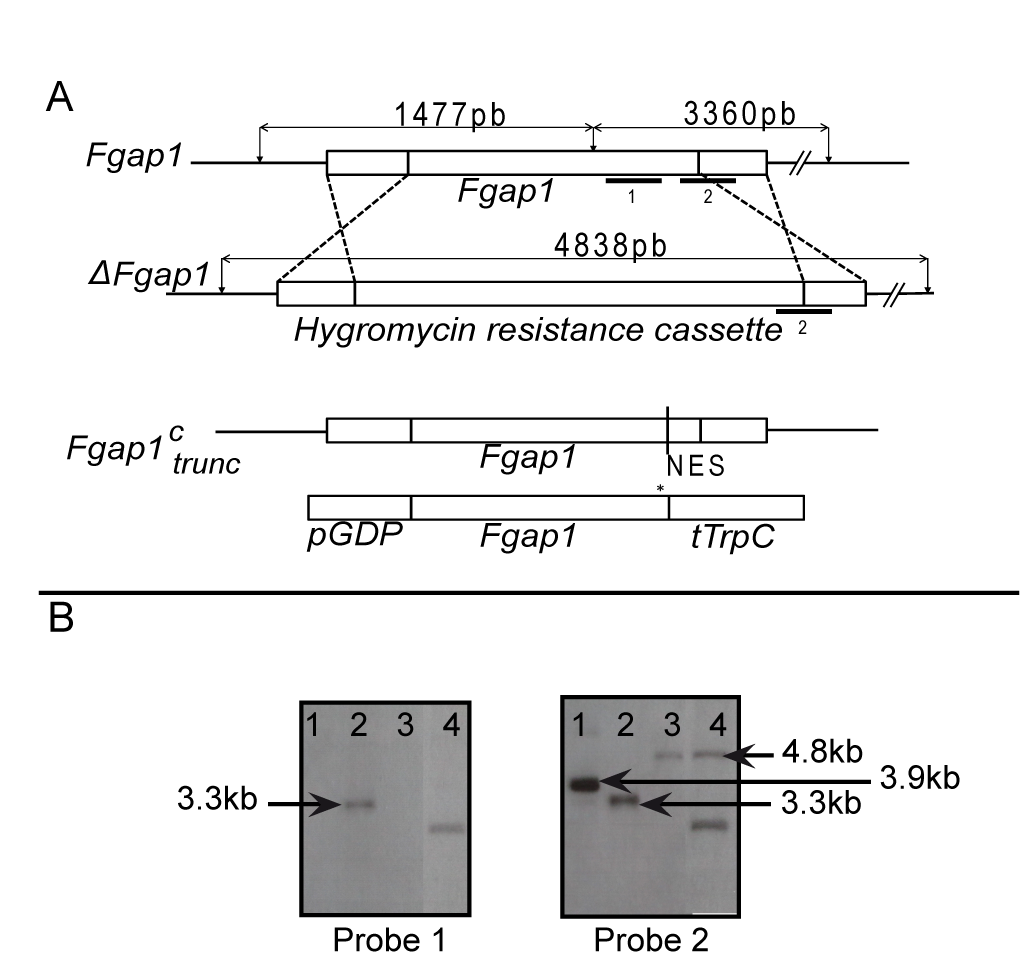


Figure S2

Supplement: Figure S2 — Construction of ΔFgap1 and Fgap1ctrunc mutants. (A) Maps of Fgap1wt locus, the ΔFgap1 construct containing the hygromycin resistance cassette and the Fgap1 c trunc construction. * corresponds to the integrated codon stop. Bars represent labeled probes 1 and 2 used for the southern blot hybridization. For the ΔFgap1 construct, among the 21 hygromycin resistant transformants, only 6 contained the replacement construct integrated at the Fgap1 locus. The transformant number 15 was selected for complementation with the wild-type copy of Fgap1. Both transformant number 15 and one of the wild-type complemented transformants were submitted to a control by Southern blot analysis, using probe 1 or 2. Vertical black arrows stand for SalI restriction sites. For the Fgap1 c trunc construct, among 27 hygromycin resistant transformants, 5 contained the truncated construction. They were cultured in GYEP medium and after 11 days of growth, 4 produced few amounts of toxins. Overexpression of Fgap1 was analyzed by Q-RT-PCR. They were analyzed by southern blot using digestion with PstI and the probe 2 to ensure that the construction was ectopic and to control the number of integrations. One transformant presented one integration and was selected for further experiments (data not shown). This transformant was analyzed by Q-RT-PCR analysis, confirming that Fgap1 was overexpressed 10 times. This mutant was called Fgap1 C trunc and retained for further experiments. (B) Southern blot hybridization. 1: pBCSKΔFgap1 (plasmid carrying the deletion construction); 2: wild-type strain; 3: ΔFgap1; 4: ΔFgap1:ap1. In the pBCSKΔFgap1, probe 2 reveals a fragment of 3.9 kb when digested with BamHI. In the wild type strain, probe 1 and 2 reveal a 3.3 kb fragment when digested with SalI. In the ΔFgap1 strain, probe 2 reveals a 4.8 kb fragment when digested with SalI. In the complemented strain ΔFgap1:ap1, probe 1 and probe 2 reveal a fragment of unknown size due to ectopic integration of the wild-type cop [file pone.0083377.s002.docx]
