# Supplementary material for: The bZIP Transcription Factor Fgap1 Mediates Oxidative Stress Response and Trichothecene Biosynthesis But Not Virulence in Fusarium graminearum
Source: PLoS One. 2013 Dec 12;8(12):e83377. doi: 10.1371/journal.pone.0083377 (PMC3861502; doi:10.1371/journal.pone.0083377)
Supplement: Table S1 — List of primers used for vector constructions and transformants screening. aCapitalized sequences in the YAPAmont-HY R and YAPAval-HY F primers correspond to the hygromycin resistance cassette to allow overlapping between the 5’UTR, the hygromycin resistance cassette and the 3’UTR. bSequences underlined in the YapAmontIFC F and YapAvalIFC R primers correspond to the multiple cloning site of the pBCSK plasmid digested with BamHI. cSequences underlined in the 5fpGPDdeb F and 3rTtrpcfin R primers correspond to the multiple cloning site of the pRS426 plasmid digested with BamHI and HindIII. dCapitalized sequences in the Yap-pGPD F and Yap-TtrpC R primers correspond to pGPD and TtrpC sequences respectively to allow overlapping between pGPD, Fgap1 and TtrpC. (DOCX) [file pone.0083377.s003.docx]

| **Oligonucleotide** | **sequence (5’ —> 3’)** |
| --- | --- |
| **Yapamont F** | **ccgtctacccaggtgtcact** |
| **Yapaval R** | **ctgcgcacgttttgtatttg** |
| **neoHY-1-fin F** | **ggccgccctgtctccggtgtccct** |
| **neoHY-8-début R** | **ctagaagaggtaaacccgaaacg** |
| **YAP-avalHY F ^a^** | **GGACACCGGAGACAGGGCGGCCaccaacaagatccggctgta** |
| **YAP-amontHY R ^a^** | **GCGTTTCGGGTTTACCTCTTCTAGggctgtgaggctcgtagaag** |
| **YapAmontIFC F ^b^** | **gcagcccgggggatccccgtctacccaggtgtcact** |
| **YapAvalIFC R ^b^** | **tagaactagtggatccctgcgcacgttttgtatttg** |
| **5fpGPDdeb F ^c^** | **aacgccagggttttcccagtcacgacgtagaattcccttgtatctctacac** |
| **3rTtrpcfin R ^c^** | **ggataacaatttcacacaggaaacagctagaattcaagagcggattcctcagtc** |
| **Yap-pGDP F ^d^** | **TATTCATCTTCCCATCCAAGAACCTTTAatggcttctaccggcactggaggcac** |
| **Yap-TtrpC R ^d^** | **GTTTGATGATTTCAGTAACGTTAAGTGtgtgccttgggacaagcttgg** |
| **pGPDyap R** | **cattaaaggttcttggatgggaagatg** |
| **TtrpC F** | **cacttaacgttactgaaatcatca** |
